# Ultrasensitive Electrochemical Detection of *Clostridium perfringens* DNA Based Morphology-Dependent DNA Adsorption Properties of CeO_2_ Nanorods in Dairy Products

**DOI:** 10.3390/s18061878

**Published:** 2018-06-08

**Authors:** Xingcan Qian, Qing Qu, Lei Li, Xin Ran, Limei Zuo, Rui Huang, Qiang Wang

**Affiliations:** 1 School of Chemical Science and Technology, Yunnan University, Kunming 650091, China; qianxc@ynu.edu.cn (X.Q.); ranzhiyong0917@163.com (X.R.); 18787593208@139.com (L.Z.); 2 Laboratory for Conservation and Utilization of Bio-Resources, Yunnan University, Kunming 650091, China; 3 Sino-Pipeline International Company Limited, Beijing 100000, China; 18487247769@139.com (R.H.); wang18782971185@163.com (Q.W.)

**Keywords:** label-free, CeO_2_ nanorods, electrochemical DNA biosensor, *Clostridium perfringens*

## Abstract

Foodborne pathogens such as *Clostridium perfringens* can cause diverse illnesses and seriously threaten to human health, yet far less attention has been given to detecting these pathogenic bacteria. Herein, two morphologies of nanoceria were synthesized via adjusting the concentration of NaOH, and CeO_2_ nanorod has been utilized as sensing material to achieve sensitive and selective detection of *C. perfringens* DNA sequence due to its strong adsorption ability towards DNA compared to nanoparticle. The DNA probe was tightly immobilized on CeO_2_/chitosan modified electrode surface via metal coordination, and the DNA surface density was 2.51 × 10^−10^ mol/cm^2^. Under optimal experimental conditions, the electrochemical impedance biosensor displays favorable selectivity toward target DNA in comparison with base-mismatched and non-complementary DNA. The dynamic linear range of the proposed biosensor for detecting oligonucleotide sequence of *Clostridium perfringens* was from 1.0 × 10^−14^ to 1.0 × 10^−7^ mol/L. The detection limit was 7.06 × 10^−15^ mol/L. In comparison, differential pulse voltammetry (DPV) method quantified the target DNA with a detection limit of 1.95 × 10^−15^ mol/L. Moreover, the DNA biosensor could detect *C. perfringens* extracted DNA in dairy products and provided a potential application in food quality control.

## 1. Introduction

Foodborne pathogens that cause numerous illnesses have become a worldwide health problem [1]. *Clostridium perfringens* is one of the most common foodborne pathogens. This predominant pathogen is a spore-forming, rod-shaped, gram-positive bacterium widely found in different environments (e.g., soils and waters) and various foods (e.g., meats and dairy products). Many countries have implemented a limit for *C. perfringens* since it is associated with two kinds of foodborne diseases: diarrhea and enteritis necroticans [2,3,4]. Thus, the development of approaches to detect *C. perfringens* is urgent and important.

Up to now, several methods for *C. perfringens* detection have been developed. Conventional methods including bacteria cultivation and biochemical tests are usually reliable and accurate but labor-consuming and time-costing [5]. In contrast, some newly developed methods based on molecule detection, such as polymerase chain reaction (PCR) [6,7], enzyme-linked immunosorbent assay (ELISA) [8], and rolling circle amplification (RCA) [9], can achieve rapid detection compared with conventional methods. Nevertheless, these methods often require complex procedures, expensive instruments and experts [10,11]. Therefore, it is significant to develop a facile, cheap and effective approach to detect *C. perfringens*.

In recent years, electrochemical DNA biosensors are widely applied to the specific detection of target DNA (tDNA) via hybridization with complementary DNA probe owing to their obvious advantages such as fast response, facilitate manipulation, low cost and high sensitivity [12]. The immobilization of DNA probe on electrode surface is an essential issue to construct an electrochemical DNA biosensor [13]. There are two types of DNA immobilization: covalent and non-covalent strategies [14]. Covalent approaches can be prepared through covalent bonding interaction, for example, Au-S bond [15], Ag-N bond [16], and amide bond [17]. However, there are some disadvantages in the application of labeled DNA due to the complex operation and expensive biochemical preparation. Contrarily, biocompatible nanomaterials are widely applied to immobilize DNA via non-covalent strategies, such as aromatic stacking [18], hydrogen bonding [19], electrostatic interaction [20], and hydrophobic force [18]. To efficiently immobilize DNA probe and decrease non-specific adsorption of DNA, the interaction between nanomaterials and DNA is more considered [21,22].

Cerium oxide (CeO_2_), an important rare earth material, was chosen for electrochemical DNA biosensor owing to its exclusive properties, such as nontoxicity, good biocompatibility, high stability and forceful absorption capability [23]. Over the years, various morphologies of CeO_2_ have been prepared, such as nanoparticles [23], nanorods [24], nanocubes [25], nanoshuttles [26], nanoplates [27], etc. and the morphology of nanoceria has a conclusive effect on its properties. Tan et al. reported that Pd/CeO_2_ nanocubes showed higher catalytic activity than octahedrons and rods [28]. Kang et al. found that the CeO_2_ nanorods have a higher adsorption capacity for fluoride compared with octahedron and nanocubes [29]. A reason that different morphologies of CeO_2_ expose different crystal planes is they display reaction activity. Therefore, it is interesting to investigate the DNA adsorption properties of CeO_2_ nanomaterials with different shapes. In addition, to enhance the sensitivity of the DNA biosensor, electrical conductivity of synthesized nanoceria should be considered. Hence, nanostructured CeO_2_ with controlled size, specific morphology, excellent conductivity and high surface charge is attracting much attention in the development of electrochemical DNA biosensor.

Chitosan (CHIT), a macromolecule polysaccharide comprising plentiful amino and hydroxyl groups, is obtained from the deacetylation of chitin. Its application in designing a DNA biosensor is becoming increasingly popular due to its biocompatibility, non-toxicity, good adhesion and attractive film-forming ability [30]. If CHIT is modified with CeO_2_ nanorods, it is possible to obtain novel functionalized materials that simultaneously have the properties of CHIT (specific surface area and electrical conductive properties) and CeO_2_ (high DNA adsorption capacity) through incorporating their individual characteristics. To our best knowledge, there is no report on employing CeO_2_/CHIT nanocomposite as sensing material to detect *Clostridium perfringens*.

**Scheme 1:**
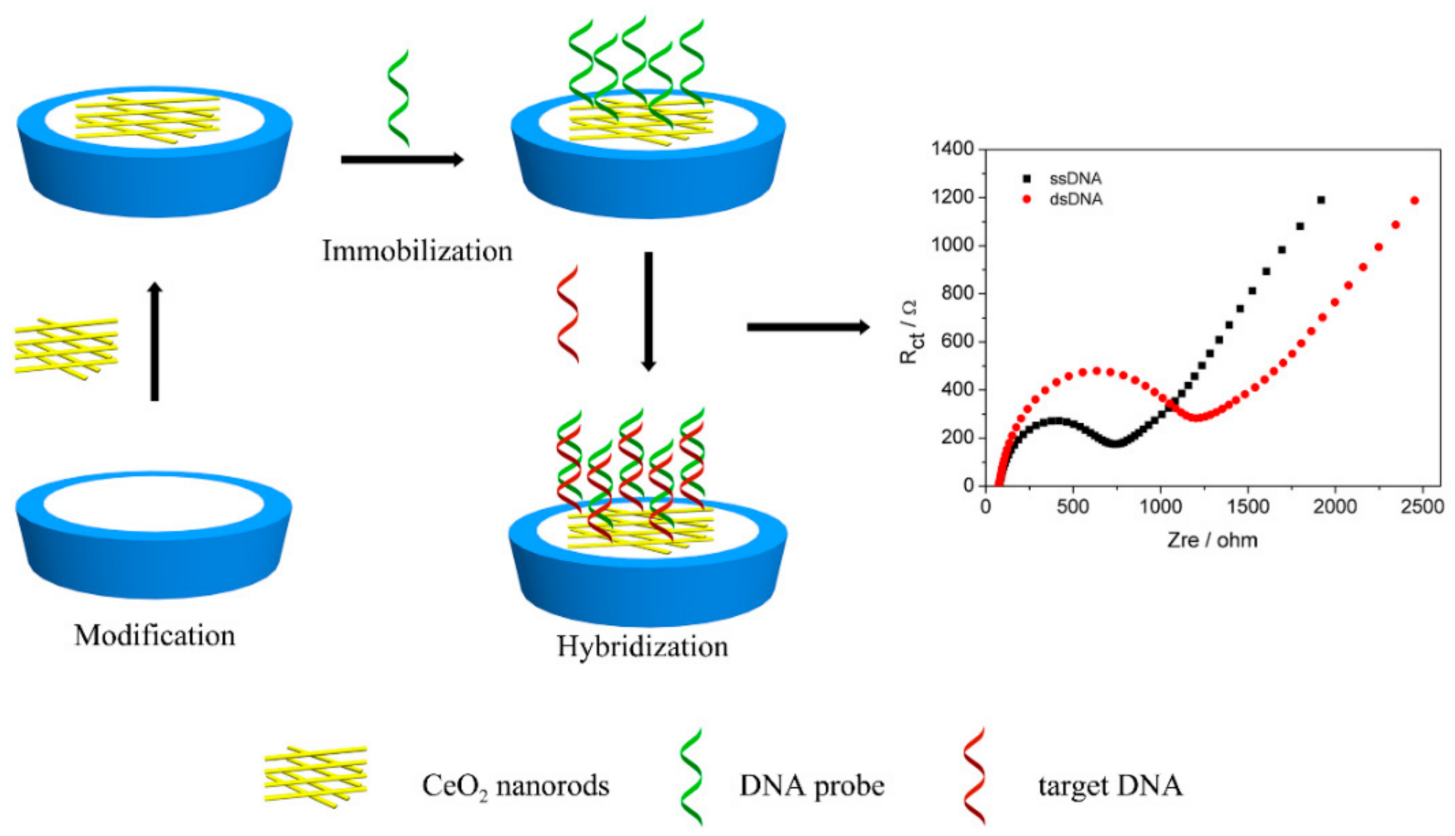
Schematic of the preparation process of the DNA biosensor.

Herein, CeO_2_ nanorods were synthesized via a simple hydrothermal method and used as sensing materials to detect *Clostridium perfringens* DNA sequence in dairy products. The rod-like CeO_2_/CHIT nanocomposite was used for immobilizing the DNA probe on the electrode surface without employing any functional groups or intermediate linker. The preparation process of the DNA biosensor is described in Scheme 1. The surface density of single-strand DNA (ssDNA) probe on the modified electrode was investigated using methylene blue (MB) as electrochemical probe. Electrochemical characterizations of the fabricated biosensor were carried out by cyclic voltammetry (CV) and electrochemical impedance spectroscopy (EIS). Under optimum conditions, the fabricated DNA biosensor exhibited high sensitivity, wide dynamic range, excellent selectivity, and satisfactory reproducibility and stability. Thus, the rod-shaped CeO_2_-based biosensor can be utilized as a potential sensing platform to detect foodborne pathogens effectively and conveniently.

## 2. Experimental

### 2.1. Materials and Reagents

Cerium nitrate hexahydrate (Ce(NO_3_)_3_·6H_2_O, 99.9%), methylene blue (MB) and 4-(2-hydroxyethyl)-1-piperazineethanesulfonic acid (HEPES) were provided by Shanghai Adamas Reagent Co., Ltd. (Shanghai, China). Chitosan (90% deacetylation degree) was purchased from Sam Chemical Technology Co., Ltd. (Guangdong, China). Tris-HCl was obtained from Beijing Solarbio Science & Technology Co., Ltd. (Beijing, China). EDTA was provided by Tianjin Zhiyuan Chemical Reagent Co., Ltd. (Tianjin, China). Trichloroacetic acid (CCl_3_COOH) and acetonitrile (CH_3_CN) were purchased from Acros Organics (Beijing, China). All other chemicals were purchased from Tianjin Fengchuan Chemical Reagent Technologies Co., Ltd. (Tianjin, China), and were of analytical reagent grade. Milli-Q ultra-pure water (18.25 MΩ cm) was used to prepare the aqueous solutions.

Sequence of *C. perfringens* gene was obtained from the National Center for Biotechnology Information (NCBI) database (accession No. NR_121697.2) [31]. Specificities of the oligonucleotide sequences were identified by Basic Local Alignment Search Tool (BLAST). The oligonucleotides used in this work were synthesized and purified by Tsingke Biological Technology Co., Ltd. (Kunming, China) with the following sequences:

ssDNA: GCT CCT TTG GTT GAA TGA TG
tDNA: CAT CAT TCA ACC AAA GGA GC
one base-mismatched DNA: CAG CAT TCA ACC AAA GGA GC
three base-mismatched DNA: CAG CAT TCA ACT AAC GGA GC
non-complementary DNA: GGC GAG CGT TAT CCG GAT TT

The stock solutions of 20-mer oligonucleotides sequence (100 µmol/L) were prepared with TE buffer (10 mmol/L Tris-HCl and 1 mmol/L EDTA, pH 8.0), and kept frozen. The hybridization solutions consisted of 10 mmol/L Tris-HCl, 1 mmol/L EDTA and 0.1 mol/L NaCl (pH 7.4).

### 2.2. Synthesis of Nanoceria

The CeO_2_ nanorods were prepared by a modified hydrothermal method [32]. Briefly, 3.84 g NaOH was dissolved in 15 mL of ultra-pure water to form a clear solution. Then, 1 mL of 0.8 mol/L Ce(NO_3_)_3_·6H_2_O aqueous solution was added drop wise into 15 mL NaOH aqueous solution under continuously stirring and the white precipitate was generated immediately. After constantly stirring for 0.5 h, the mixed solution was transferred into an autoclave and heated at 100 °C for 24 h. The white products after hydrothermal treatment were washed by ultra-pure water and ethanol alternatively several times, and dried at 60 °C overnight. The CeO_2_ nanoparticles were prepared through the same methods at 100 °C for 24 h, while *C*_NaOH_ was 0.01 mol/L.

### 2.3. Apparatus and Characterization

The synthesized samples were characterized by X-ray powder diffraction (XRD) performed on a Bruker D8-Advance diffractometer with Cu Kα radiation (λ = 0.154 nm) in the 2θ range from 10° to 90°. Morphologies of obtained nanoparticles and nanorods were studied by transmission electron microscope (TEM, JEM-2100, JEOL Ltd., Tokyo, Japan). The ultraviolet-visible light (UV–vis) absorption spectra were recorded using a spectrophotometer (U-2001 Hitachi, Tokyo, Japan) and the analyzed range was 200–800 nm. X-ray photoelectron spectroscopy (XPS) was recorded by a K-Alpha^+^ (Thermo Fisher Scientific, Waltham, MA, USA) operating with Mono Al Kα radiation. The values of zeta potential were measured using a Malvern Zeta Sizer Nano (Malvern Instruments Ltd., Worcestershire, UK).

### 2.4. Preparation of the CeO_2_/CHIT/GCE

CHIT solution (2.0 mg/mL) was prepared by dissolving chitosan (100 mg) in 50 mL aqueous solution containing 1.0% acetic acid. CeO_2_ nanorods (5 mg) were stirred and sonicated in the CHIT solution for 30 min at room temperature to form a highly dispersed colloidal solution.

Before each experiment, a glassy carbon electrode (GCE) was sequentially polished with 0.3 µm and 0.05 µm Al_2_O_3_ powders, respectively. Then, it was cleaned ultrasonically in ultra-pure water and ethanol for 3 min. The CeO_2_/CHIT composite (7 µL) was dropped onto electrode surface and dried in air. The CHIT/GCE and CeO_2_/GCE were carried out through similar procedure.

### 2.5. DNA Probe Immobilization and Hybridization

Five microliters of ssDNA probe (1.0 × 10^−7^ mol/L) solution was pipetted onto the CeO_2_/CHIT electrode surface and dried at 25 °C for 30 min. Then, the ssDNA/CeO_2_/CHIT/GCE was rinsed with ultra-pure water to remove non-specific adsorption of DNA. The hybridization reaction was conducted by dropping 5 µL of tDNA solution onto the surface of ssDNA/CeO_2_/CHIT electrode and the reaction was kept at 50 °C for 30 min. Then, the dsDNA/CeO_2_/CHIT/GCE was rinsed with water to prevent unhybridized tDNA. The hybridization of ssDNA probe with one base-mismatched DNA, three base-mismatched DNA and non-complementary DNA were performed through similar procedure.

### 2.6. Electrochemical Measurements

Cyclic voltammetry (CV) and electrochemical impedance spectroscopy (EIS) experiments were performed by utilizing a CHI 660E Electrochemical Workstation (Shanghai Chenhua Instrument) and connected to a three-electrode system including a modified CeO_2_/CHIT/GCE as a working electrode, a platinum wire served as counter electrode, and a saturated calomel reference electrode, which were performed in 0.1 mol/L sodium phosphate buffer (PBS, pH 7.4) containing 2.0 mmol/L [Fe(CN)_6_]^3−/4−^ and 0.1 mol/L KCl. CV measurements was scanned between −0.2 V and 0.5 V (vs. SCE) at a scan rate of 0.05 V/s. The frequencies of EIS measurements were recorded from 0.1 Hz to 10 KHz at the amplitude of 5 mV. All electrochemical experiments were conducted in triplicate.

### 2.7. Real Sample Assay

*Clostridium perfringens* (ATCC 13124) was provided by Guangdong Huankai Microbial Sci. & Tech. Co., Ltd. (Guangdong, China). The bacteria were grown in cooked meat medium for 15 h at 37 °C. The DNA extraction process was carried out using a kit manufacturer (Tiangen Biotech Co., Ltd., Beijing, China). The DNA extraction efficacy was estimated by measuring absorbance at 260 nm using an ultraviolet spectrophotometer.

The pure milk and milk powder were purchased from a local supermarket. According to the methods reported previously [33], 1 mL of CCl_3_COOH, 1 mL of CH_3_CN, 7 mL (for pure milk)/9 mL (for milk powder) of ultra-pure water were added into 2 mL of pure milk or 1 g of milk powder. Then, the mixture was ultrasonically extracted for 0.5 h and centrifugated at 11,500× *g* for 10 min to eliminate protein. The resultant supernatant was adjusted to pH 7.40 with PBS and then used to prepare different concentrations of *C. perfringens* gene from (1 to 50 pmol/L). Before the hybridization process, DNA sequence was denatured at 95 °C for 5 min into ssDNA. Detection process was performed under the optimum conditions.

## 3. Results and Discussion

### 3.1. Morphological and Structural Characterization

**Figure 1:**
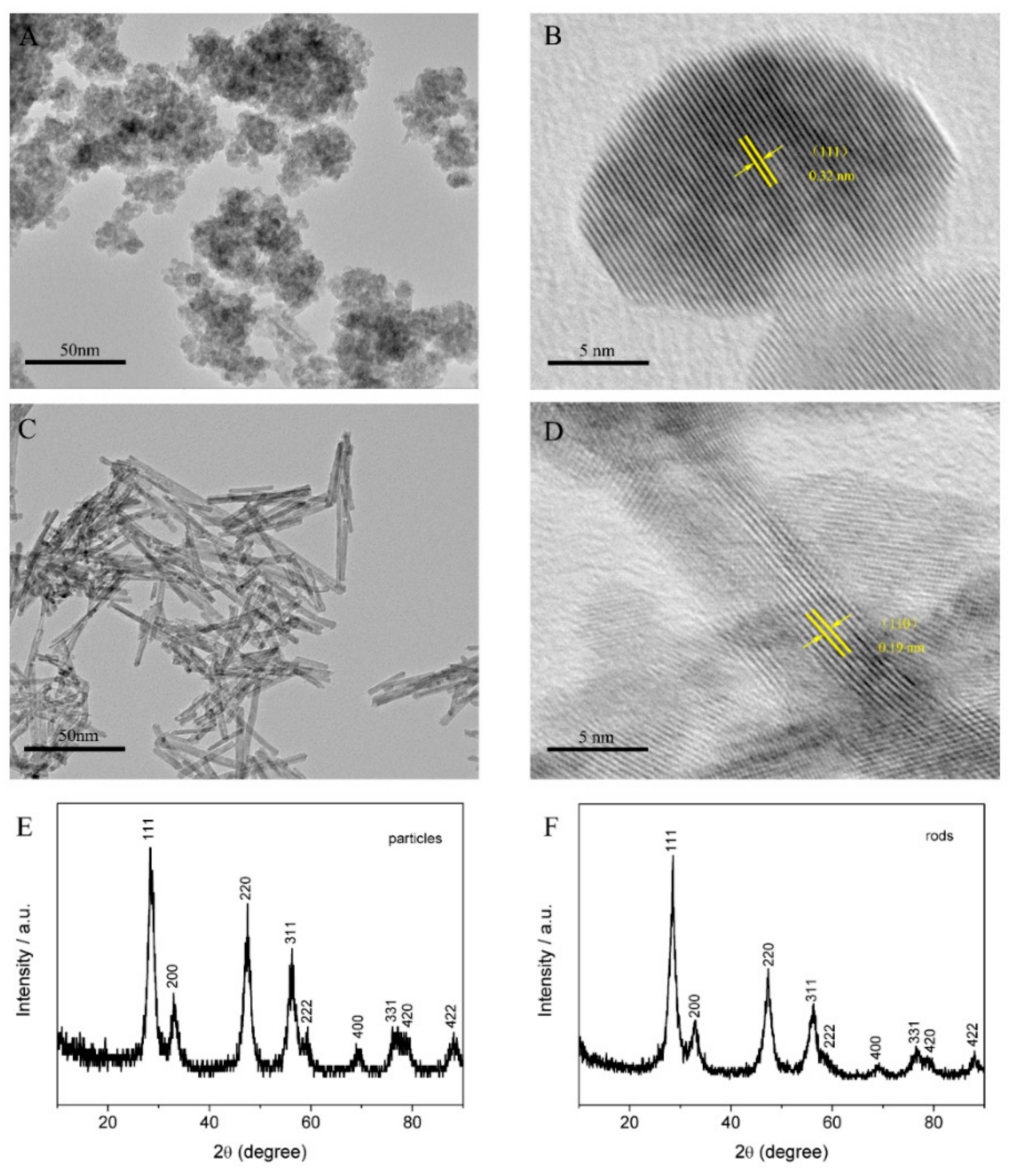
TEM and HRTEM images of CeO_2_: nanoparticles (**A**,**B**); and nanorods (**C**,**D**). XRD pattern of CeO_2_: nanoparticles (**E**); and nanorods (**F**).

The morphologies of the synthesized nanoceria were examined by TEM technique. Figure 1A displays the as-obtained uniform CeO_2_ nanoparticles in the size of 10.2 ± 1.2 nm. As shown in Figure 1B, the high-resolution TEM (HRTEM) image showed that the clear lattice fringes with *d*-spacing of 0.32 nm are corresponding to {111} crystal plane. With increasing NaOH concentration, the obtained products possessed an obvious rod-like morphology (Figure 1C), with 100 nm in length and 6–10 nm in width. Figure 1D exhibits the lattice spacing of nanorods was 0.19 nm, belonging to the {110} planes. In brief, the prepared CeO_2_ nanoparticles and nanorods present different crystallographic planes of {111} and {110}, which may possess distinct physicochemical properties, and therefore display different DNA adsorption properties.

XRD analysis was performed to investigate the crystal structure of the as-synthesized products. Figure 1E,F shows the XRD pattern of CeO_2_ nanoparticles and nanorods, respectively. All detectable peaks corresponded to a typical fluorite structure (JCPDS No. 34-0394, space group Fm-3m), suggesting that nanoceria was successfully synthesized [34].

### 3.2. The Surface State of the Synthesized Nanoceria

**Figure 2:**
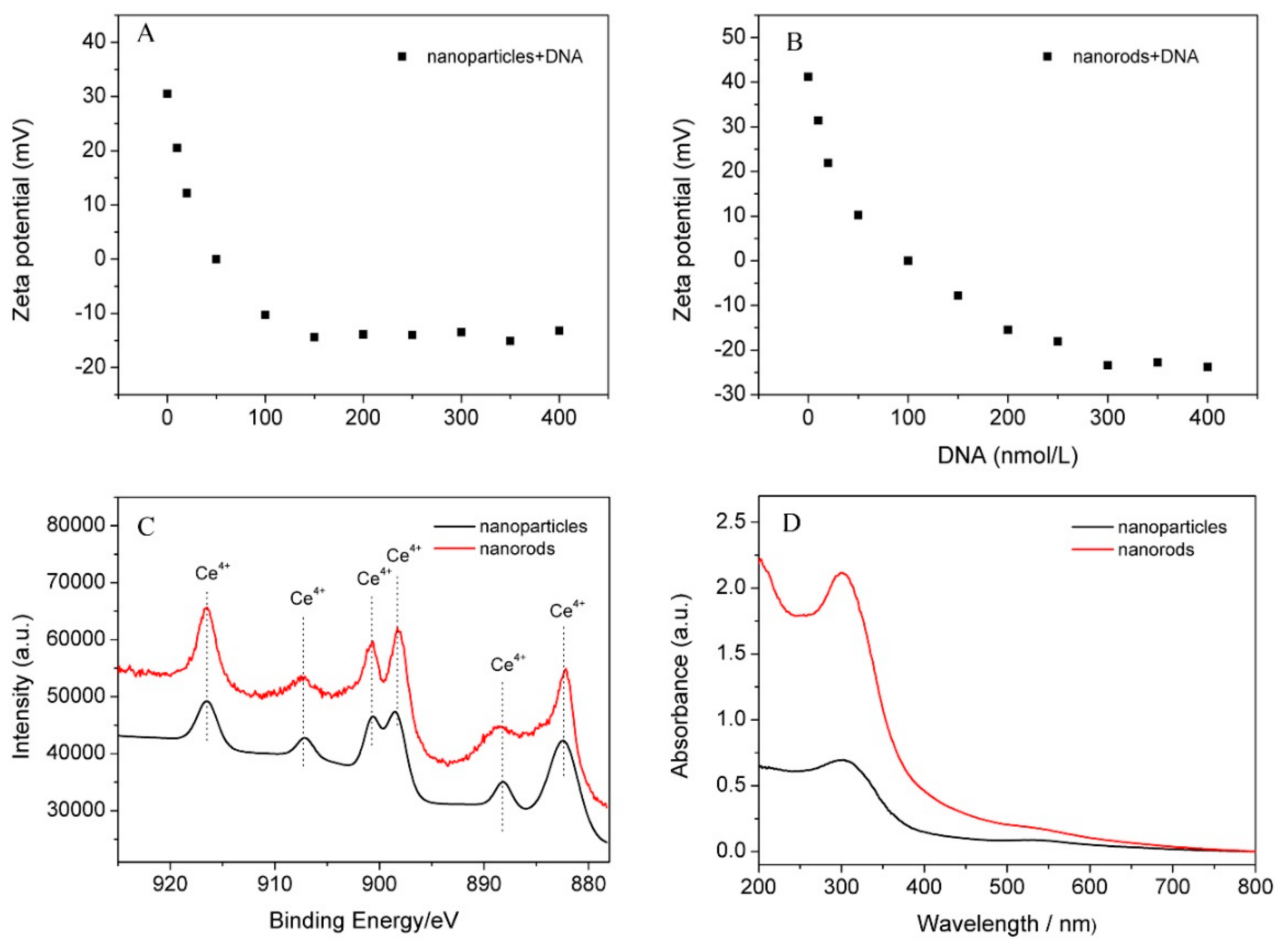
The change of zeta potential from positive to negative CeO_2_: (**A**) nanoparticles; and (**B**) nanorods; (**C**) Ce 3d XPS spectrum of nanoparticles and nanorods; and (**D**) UV–vis adsorption spectra of nanoceria.

To adsorb DNA probe, the surface charge of nanoceria is significant. The zeta potential of CeO_2_ nanoparticles and nanorods were measured, as shown in Figure 2. The average zeta potentials of nanoparticles and nanorods without adsorption of DNA were 30.5 mV and 41.2 mV, respectively, indicating that both had positive surface charge. The nanoparticles and nanorods exhibited excellent stability and dispersity, which was because the zeta potential values were higher than 30 mV [35]. Moreover, the zeta potential of CeO_2_ nanorods was approximately 10 mV higher compared with that of nanoparticles, suggesting that a higher surface positive charge is present.

Moreover, the effect of DNA adsorption on the zeta potential of nanoceria were investigated. According to the literature [36], CeO_2_ nanoparticles and nanorods were incubated with different concentrations of DNA probe (10, 20, 50, 100, 150, 200, 250, 300, 350, and 400 nmol/L) in HEPES buffer (pH = 7.4, 10 mmol/L) containing 0.1 mol/L NaCl at 37 °C for 30 min, respectively. With increasing concentration of DNA, the zeta potential of nanoceria changed from positive to negative (Figure 2A,B), illustrating that DNA was coating on the surface of nanoparticles and nanorods. Additionally, CeO_2_ nanorods exhibited stronger adsorption capacity (~300 nmol/L) than nanoparticles (~150 nmol/L), demonstrating that the interaction between nanorods and DNA are much stronger than nanoparticles. Thus, besides electrostatic adsorption, metal coordination might exist [37].

To confirm this inference, the surface valence of nanoceria was estimated using XPS. As shown in Figure 2C, the Ce 3d XPS spectra can be deconvoluted into six peaks, corresponding to 916.4, 907.2, 900.7, 898.0, 888.6 and 882.2 eV. The characteristic satellite peak of Ce^4+^ at 916.4 eV suggested that the main valences of cerium in two samples were +4 [38]. Compared to nanoparticle, CeO_2_ nanorods presented higher peak intensity, suggesting that they had a higher Ce^4+^ concentration.

To identify the chemical state of nanoceria, UV–vis absorption spectroscopy was used. Generally, the 300–400 nm spectral range, which attributes to the cerium (IV) concentration, could show a corresponding increase along with the increment of Ce^4+^ [39]. UV–vis absorption spectra of nanoceria is illustrated in Figure 2D. Obviously, the main valence state of nanoparticles and nanorods was +4. The maximum absorption peak was ascribed to CeO_2_ nanorods, showing that nanorods have a higher surface Ce^4+^ concentration than nanoparticles. The results are consistent with zeta potential measurements.

Based on above-mentioned analysis, CeO_2_ nanorods present better DNA adsorption performance than nanoparticles. CeO_2_ nanorods possess high aspect-ratio structure, excellent stability and dispersity, and high surface Ce^4+^ concentration, exhibiting higher DNA adsorption capacity than nanoparticles. Additionally, the predominant exposed {110} planes with high surface energy of CeO_2_ nanorods [40] can provide reactive sites for metal coordination interaction between Ce^4+^ ions and DNA. Therefore, CeO_2_ nanorods were selected as sensing materials to detection of *Clostridium perfringens* DNA oligonucleotide sequence.

### 3.3. Electrochemical Characterization of Biosensor

**Figure 3:**
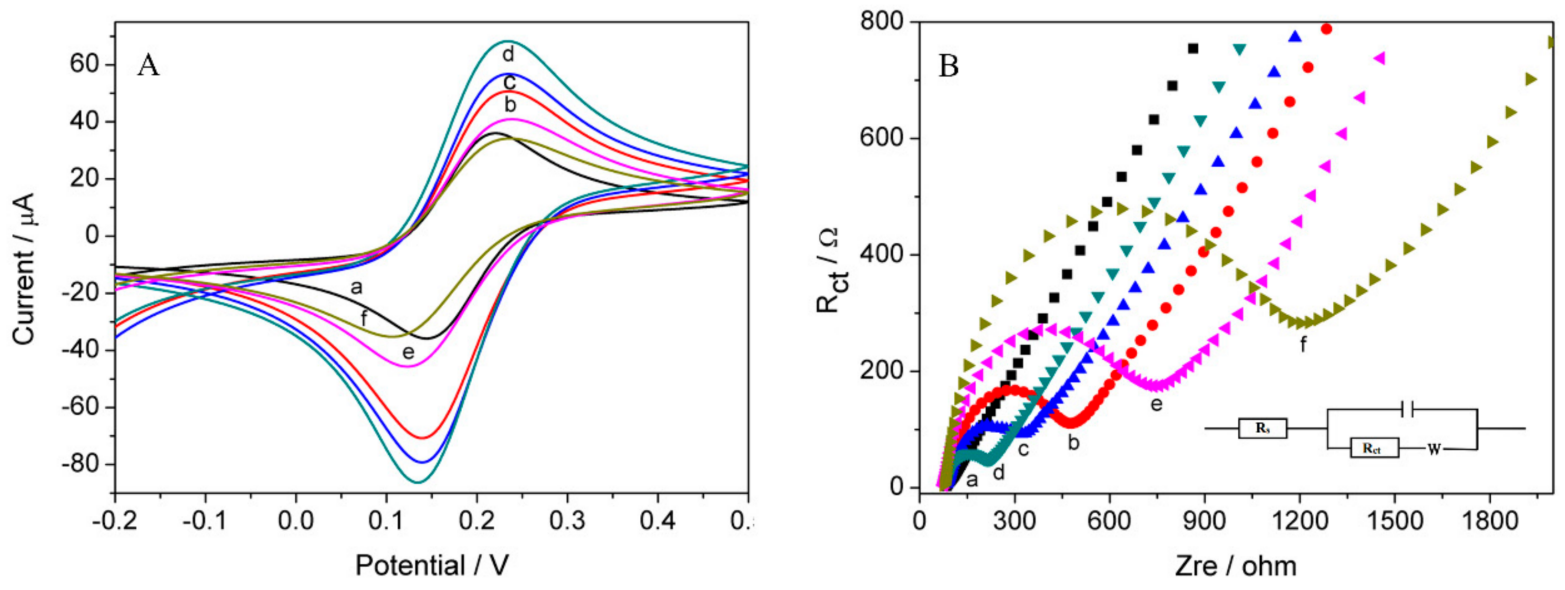
(**A**) CVs; and (**B**) Nyquist diagrams in 0.1 M PBS (pH 7.4) containing 2.0 mmol/L [Fe(CN)_6_]^3−/4−^ and 0.1mol/L KCl at: (a) GCE; (b) CHIT/GCE; (c) CeO_2_/GCE; (d) CeO_2_/CHIT/GCE; (e) ssDNA/CeO_2_/CHIT/GCE; and (f) dsDNA/CeO_2_/CHIT/GCE. Inset is the equivalent circuit.

The electrochemical performance of the prepared electrodes was tested by CV measurements. As shown in Figure 3A, a pair of well-defined redox peaks could be discovered in all CV plots. The current responses of CHIT/GCE (Figure 3Ab 50.7 µA) increased markedly compared to those of GCE (Figure 3Aa 36.0 µA), contributing to the cationic amino groups on CHIT combined with negative charged ferricyanide ions through electrostatic interaction [41]. The peak current at CeO_2_/GCE (Figure 3Ac 56.8 µA) showed that the synthesized CeO_2_ nanorods had an excellent electronic conductivity. The response signal of CeO_2_/CHIT/GCE (Figure 3Ad 68.2 µA) increased in comparison to CHIT/GCE and CeO_2_/GCE, which could contribute to the amplification effect of CeO_2_ nanorods and chitosan, in which the rod-like CeO_2_ enhances electron transfer and CHIT provides a large electroactive surface area to facilitate diffusion of ferricyanide ions [31]. After immobilization of the DNA probe on CeO_2_/CHIT/GCE, the current response decreased dramatically. This is because of the electrostatic repulsion interaction between [Fe(CN)_6_]^3−/4−^ and negatively charged ssDNA [42]. The peak current of dsDNA/CeO_2_/CHIT/GCE (Figure 3Af 34.2 µA) decreased notably as compared with that at ssDNA/CeO_2_/CHIT/GCE (Figure 3Ae 40.9 µA), demonstrating that sufficient negative phosphate groups and large space steric effect made the interfacial electron transfer more difficult.

It is well known that EIS is an effective technique to study interfacial properties of the modified electrode surface. The impedance spectra comprise a semicircle portion at higher frequencies representing the charge transfer-resistance (R_ct_) and a linear portion at lower frequencies relating to the diffusion process. Figure 3B presents the Nyquist diagrams of: GCE (a); CHIT/GCE (b); CeO_2_/GCE (c); CeO_2_/CHIT/GCE (d); ssDNA/CeO_2_/CHIT/GCE (e); and dsDNA/CeO_2_/CHIT/GCE (f). Besides, electrochemical parameters of different modified electrodes are listed in Table 1. The bare GCE displayed an almost straight line, implying that the charge transfer was a diffusion-controlled process, and the R_ct_ value of GCE was relatively high [43]. The electrode modified with chitosan showed a lower resistance, which indicated that CHIT film improved conductivity of the electrode. After the modification of CeO_2_ nanorods, the R_ct_ declined, which implied that nanorods is an outstanding electric conducting material. The R_ct_ value of CeO_2_/CHIT/GCE decreased dramatically due to the signal amplification action based on nanorods and CHIT. The R_ct_ increased evidently because DNA probe loaded on the surface of CeO_2_/CHIT modified electrode. Enhancement R_ct_ was found after hybridization of DNA probe with tDNA, exhibiting that the formation of double-stranded DNA on the modified electrode [44].

### 3.4. Electrochemical Studies of DNA Immobilization

**Figure 4:**
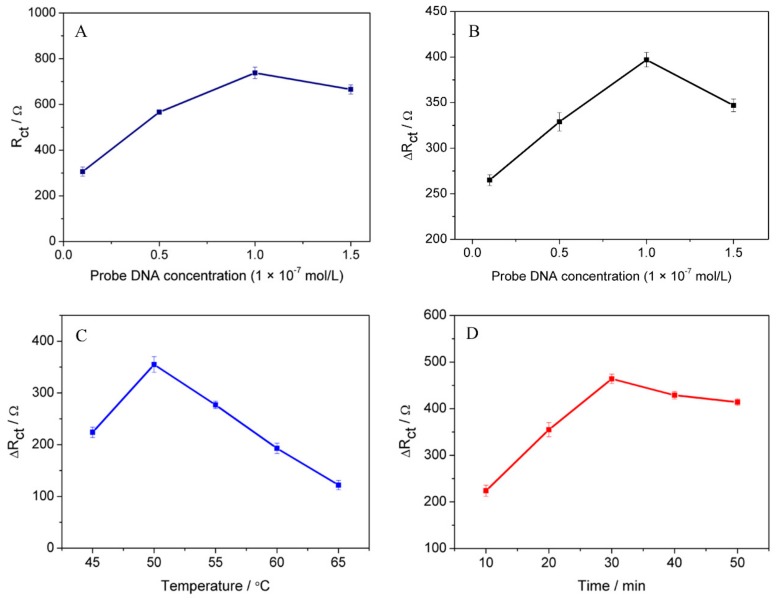
(**A**) Influence of different concentratiosn of ssDNA probe at the CeO_2_/CHIT/GCE; (**B**) the comparison of different concentratiosn of ssDNA probe hybridized with 1 × 10^−7^ tDNA; (**C**) influence of different hybridization temperatures; and (**D**) influence of different hybridization periods at 50 °C. The error bars represent the standard deviation of three experiments.

Owing to the amount of immobilized ssDNA substantially impacting the sensitivity of the biosensor, the influence of ssDNA concentration (0.1, 0.5, 1.0, and 1.5 × 10^−7^ mol/L) on the response of the fabricated biosensor was examined. As Figure 4A illustrates, the R_ct_ gradually increased with increasing ssDNA concentration to 1.0 × 10^−7^ mol/L and then decreased. Therefore, we used DNA probe at a concentration of 1.0 × 10^−7^ mol/L in this work.

In the process of ssDNA concentration optimization, the comparison experiments of the charge resistance difference obtained after and before hybridization were also examined. To explore the ssDNA concentration with the best sensitivity more easily, the concentration of tDNA was reasonably set to 1 × 10^−7^ mol/L. In Figure 4B, the maximum charge resistance difference (ΔRct = (R_ct_)dsDNA–(R_ct_)ssDNA) was obtained at the ssDNA concentration of 1 × 10^−7^ mol/L, indicating that the concentration of ssDNA immobilized on the electrode with the best sensitivity and the optimal hybridization efficiency was 1 × 10^−7^ mol/L [45].

The surface densities of oligonucleotide probe on differently modified electrodes were investigated according to the method reported [46]. Based on the fundamental principles of this method, the molar quantity of methylene blue (MB) was calculated from the cyclic voltammetry, by use of the following equation: *N* = *Q*/(*neN*_A_), where *N* means the molar quantity of MB; *Q* equals the electric charge quantity of MB; *n* represents the numbers of electrons engaged in MB redox reaction, in this experiment, *n* = 2; *e* is the electric charge on single electron, and its value is 1.60 × 10^−19^ C; and *N*_A_ is Avogadro’s constant, which is 6.02 × 10^23^ mol^−1^. In our experiment, we received the electric charge quantity of MB by integrating its cathodic peak area after combination with ssDNA probe on differently modified electrode. Therefore, *N* was calculated to be 8.31 × 10^−11^ mol for the ssDNA/CHIT/GCE, 9.06 × 10^−11^ mol for the ssDNA/CeO_2_/GCE and 1.06 × 10^−10^ mol for the ssDNA/CeO_2_/CHIT/GCE. One guanine base (G) can combine with one MB molecule, and every *C. perfringens* ssDNA probe contains six G bases. The surface density of ssDNA probe was therefore calculated as the product of the molar quantity of MB and the stoichiometric ratio between MB and DNA probe (6:1). In this experiment, the geometric area of every GCE (3 mm in diameter) was 7.07 × 10^−2^ cm^2^, and the surface density of oligonucleotide probe on the CeO_2_/CHIT/GCE was calculated to be 2.51 × 10^−10^ mol/cm^2^, which was higher than that on the CHIT/GCE (1.96 × 10^−10^ mol/cm^2^) and CeO_2_/GCE (2.14 × 10^−10^ mol/cm^2^). The results demonstrated that the combination of the CeO_2_/CHIT nanocomposite membrane could enormously enhance the loading of oligonucleotide probe on the electrode surface, and therefore improve the sensitivity of the biosensor.

### 3.5. Optimization of DNA Hybridization

The hybridization temperature has significant influence on the hybridization reaction. After the immobilization of ssDNA under the optimal concentration, 5 µL tDNA (1.0 × 10^−7^ mol/L) was dropped onto ssDNA/CeO_2_/CHIT electrode at different temperatures: 45, 50, 55, 60 and 65 °C. The results of this investigation are given in Figure 4C. As the results showed, the ΔR_ct_ value of biosensor increased as the hybridization temperature increased up to 50 °C. Above 50 °C, the R_ct_ gradually decreased [15]. Therefore, we used 50 °C as the optimum hybridization temperature.

Another important parameter which can affect the hybridization reaction is time. As Figure 4D illustrates, the ΔR_ct_ value constantly increased with increasing the time to 30 min and then the response signal remained steady, indicating that the optimal hybridization time was 30 min [47]. Therefore, 50 °C and 30 min were selected as hybridization temperature and time.

### 3.6. Quantitative Analysis of C. perfringens Sequence

**Figure 5:**
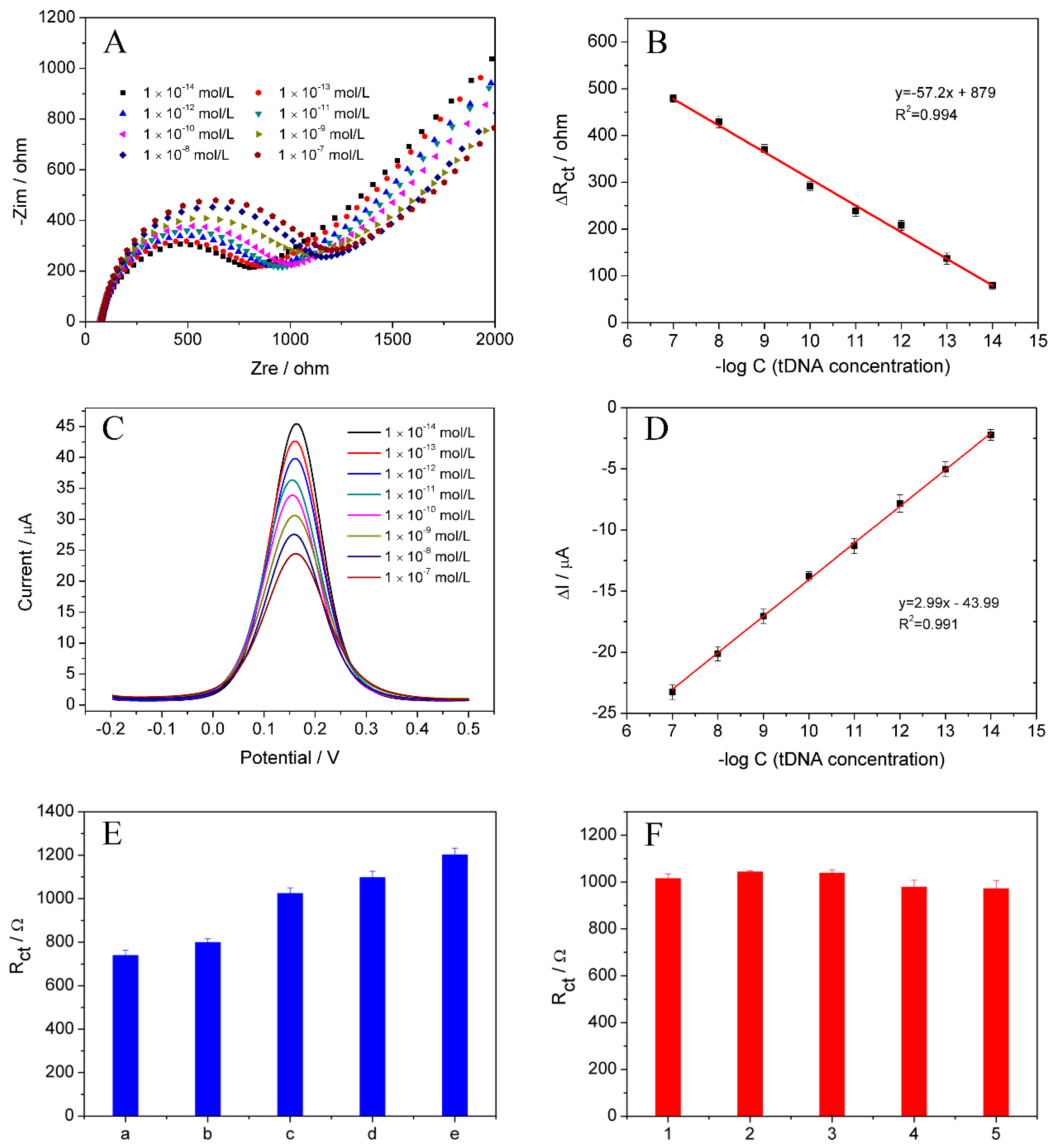
(**A**) Resistance values of the biosensor containing increasing concentration of C. perfringens sequence (1.0 × 10^−7^, 1.0 × 10^−8^, 1.0 × 10^−9^, 1.0 × 10^−10^, 1.0 × 10^−11^, 1.0 × 10^−12^, 1.0 × 10^−13^, and 1.0 × 10^−14^ mol/L). (**B**) Plots of ΔR vs. logarithm of tDNA concentration. (**C**) DPV responses for different concentration of tDNA from 1.0 × 10^−7^ to 1.0 × 10^−14^ mol/L. (**D**) Plots of ΔI vs. logarithm of tDNA concentration. (**E**) Comparison of R_ct_ values of: ssDNA (a); ssDNA hybridized with non-complementray DNA (b); three base-mismatched DNA (c); one base-mismatched DNA (d); and target DNA (e). (**F**) Differences of five separate electrode. The error bars represent the standard deviation of three experiments.

Under the optimum conditions, the quantitative analysis of the *C. perfringens* sequence was investigated using the ssDNA to hybridize with different concentration of tDNA. In Figure 5A, the EIS values increased as the concentration of tDNA increased. As shown in Figure 5B, the response signal (ΔR_ct_) had a good linear relationship with the logarithm of the tDNA concentrations in the range of 1.0 × 10^−14^ to 1.0 × 10^−7^ mol/L. The regression equation was ΔR = −57.2 × logC + 879 (C, mol/L; ΔR_ct_, Ω) with a correlation coefficient of 0.994, and a detection limit of 7.06 × 10^−15^ mol/L could be evaluated using 3 s/m formula according to the reported literature [48], where “s” represents the standard deviation of the blank solution and “m” is the slope of the linear calibration graph between electrochemical resistance and analyte concentration. To demonstrate the analytical performance of this sensing platform, DPV measurements were also utilized to quantitatively determine *C. perfringens* DNA in 0.1 mol/L PBS containing 2.0 mmol/L [Fe(CN)_6_]^3−/4−^ and 0.1 mol/L KCl. In Figure 5C,D, with increasing concentration of tDNA, the DPV response declined, which revealed a good linear relationship between logC_tDNA_ and current in the range of 1.0 × 10^−14^ ~ 1.0 × 10^−7^ mol/L (R^2^ = 0.991). The linear equation and the detection limit were ΔI = 2.99 × logC − 43.99 (ΔI = I_dsDNA_ − I_ssDNA_) and 1.95 × 10^−15^ mol/L (3 s/m), respectively. Both methods (EIS and DPV) obtained satisfying detection limits, indicating that the sensing platform possessed an outstanding analytical property. Compared to some previous works (Table 2), the proposed biosensor displayed higher sensitivity, which might be attributed to that CeO_2_ nanorods had the larger loading capability towards ssDNA and could make the immobilized probes possess more reasonable orientation and spacing between them [49].

### 3.7. The Selectivity of DNA Biosensor

The selectivity of the fabricated biosensor was examined by comparing the R_ct_ value before and after hybridization with one base-mismatched DNA, three base-mismatched DNA and non-complementary DNA. The resultant histograms are given in Figure 5C. It is obvious that only a negligible change was discovered at ssDNA/CeO_2_/CHIT/GCE before and after hybridization with non-complementary DNA (Figure 5Ea,b). The results displayed that there was no hybridization reaction. The signal increased noticeably when the ssDNA was hybridized with the tDNA (Figure 5Ee), suggesting that the double stranded DNA was formed on the electrode [51]. After the ssDNA was hybridized with one base-mismatched DNA (Figure 5Ed) or three base-mismatched DNA (Figure 5Ec), the signal was much smaller than that of dsDNA/CeO_2_/CHIT/GCE. The result revealed that the biosensor had a good performance to identify the target DNA on ssDNA/CeO_2_/CHIT/GCE.

### 3.8. The Producibility, Stability, and Regeneration Ability of DNA Biosensor

The reproducibility of the proposed biosensor was investigated using EIS method to detect the tDNA (1.0 × 10^−10^ mol/L). In Figure 5F, five parallel-modified electrodes under the same conditions were: 1015 (a); 1043 (b); 1038 (c); 978 (d); and 972 Ω (e). The relative standard deviation (RSD) was 3.27% (*n* = 5). The results showed that the satisfactory reproducibility of the fabricated DNA biosensor.

The stability of the prepared biosensor was also examined. The ssDNA/CeO_2_/CHIT/GCE could be stored at 4 °C for 20 days and the decrease of the R_ct_ value was 5.30%. The results demonstrated that the fabricated biosensor had high stability.

The regeneration ability of the DNA biosensor was tested according to the literature method [52]. In this experiment, the double stranded DNA on the CeO_2_/CHIT electrode was denatured by immersing the modified electrode into a 0.2 mol/L NaOH solution for 5 min and then washed with ultra-pure water. The signal responses were recorded using EIS method. Our test indicated only a 3.47% decrease in the first cycle, and the following experiment displayed that the biosensor could be reproduced for four times under continuously hybridization and denaturation, indicating an excellent regeneration ability.

### 3.9. Detection of tDNA in Dairy Products

To identify the performance of the fabricated biosensor in the real sample, the standard addition method was employed to analyze content of *C. perfringens* DNA in the dairy products, including pure milk and milk powder. As shown in Table 3, it could be observed that the RSD was lower than 4.96% and the average recoveries of *C. perfringens* DNA in dairy products ranged from 95.4 to 102.6%. The obtained results indicated that the proposed biosensor with strong anti-interference capability, which could be applied to detect *C. perfringens* in real samples.

## 4. Conclusions

In summary, crystalline, rod-like CeO_2_ nanorods with high aspect ratio were synthesized and utilized as sensing materials to fabricate a simple, label-free, sensitive and selective electrochemical DNA biosensor for detection of *C. perfringens*. Under appropriate conditions, the CeO_2_/CHIT nanocomposite-based DNA biosensor detected *C. perfringens* DNA oligonucleotides sequence in liner range of 1.0 × 10^−14^ to 1.0 × 10^−7^ mol/L with detection limit of 7.06 × 10^−15^ mol/L. The relative standard deviation for five separate DNA biosensors was 3.27%. Furthermore, the fabricated biosensor could maintain performances in low temperature and regenerating for four times after denaturing. The fabricated biosensor was applied for the determination of *C. perfringens* DNA sequence in dairy products with RSD lower than 4.96%. The attractive characteristics of easy-to-operate process, high sensitivity and low cost gives the biosensor potential for foodborne pathogenic bacteria detection.

## Figures and Tables

**Table 1:** Electrochemical parameters of different modified electrodes were obtained using EIS measurements in 0.1 mol/L PBS (pH 7.4) containing 2.0 mmol/L [Fe(CN)_6_]^3−/4−^ and 0.1 mol/L KCl (vs. SCE).

Electrode	Rs (Ω)	Rct (Ω)
GCE	81.20 (1.095)	979.9 (1.780)
CHIT/GCE	76.06 (0.9024)	373.8 (1.214)
CeO_2_/GCE	80.15 (1.253)	270.6 (2.627)
CeO_2_/CHIT/GCE	74.31 (1.100)	166.6 (1.704)
ssDNA/ CeO_2_/CHIT/GCE	76.08 (0.6078)	629.2 (0.8229)
dsDNA/ CeO_2_/CHIT/GCE	76.01 (0.6954)	1088 (0.8843)

**Table 2:** Comparison of the CeO_2_/CHIT nanocomposite-based DNA biosensor with some previous works for detection *Clostridium perfringens.*

Modified Material and Electrode	Detection Technique	Liner Range (mol/L)	Detection Limit (mol/L)	References
AuNPs/graphene/GCE	DPV	10^−12^–10^−6^	10^−12^	[50]
Au	ECL	10^−15^–10^−9^	10^−15^	[9]
CeO2/CHIT/GCE	EIS	10^−14^–10^−7^	10^−15^	this work

**Table 3:** Detection of *Clostridium perfringens* in dairy products by CeO_2_/CHIT nanocomposite-based DNA biosensor.

Samples	Added (pmol/L)	Found (pmol/L)	Recovery (%)	RSD (%)
pure milk	10	9.77	97.7	1.66
	20	19.9	99.5	2.74
	50	51.3	102.6	4.96
milk powder	10	9.54	95.4	2.53
	20	20.4	102	3.25
	50	48.9	97.8	3.45
